# Uncovering potential diagnostic biomarkers of acute myocardial infarction based on machine learning and analyzing its relationship with immune cells

**DOI:** 10.1186/s12872-022-02999-7

**Published:** 2023-01-04

**Authors:** Ling Kang, Qiang Zhao, Ke Jiang, Xiaoyan Yu, Hui Chao, Lijuan Yin, Yueqing Wang

**Affiliations:** 1 Department of Cardiology, The Second Affiliated Hospital of Shandong First Medical University, No. 706, Taishan Street, Taian, 271000 Shandong China; 2 Coronary Care Unit, The Second Affiliated Hospital of Shandong First Medical University, No. 706, Taishan Street, Taian, 271000 Shandong China

**Keywords:** Acute myocardial infarction, Biomarker, Machine learning, Functional enrichment, Differentially expressed mRNAs, miRNA-mRNA network

## Abstract

**Background:**

Acute myocardial infarction (AMI) is a common cardiovascular disease. This study aimed to mine biomarkers associated with AMI to aid in clinical diagnosis and management.

**Methods:**

All mRNA and miRNA data were downloaded from public database. Differentially expressed mRNAs (DEmRNAs) and differentially expressed miRNAs (DEmiRNAs) were identified using the metaMA and limma packages, respectively. Functional analysis of the DEmRNAs was performed. In order to explore the relationship between miRNA and mRNA, we construct miRNA-mRNA negative regulatory network. Potential biomarkers were identified based on machine learning. Subsequently, ROC and immune correlation analysis were performed on the identified key DEmRNA biomarkers.

**Results:**

According to the false discovery rate < 0.05, 92 DEmRNAs and 272 DEmiRNAs were identified. GSEA analysis found that kegg_peroxisome was up-regulated in AMI and kegg_steroid_hormone_biosynthesis was down-regulated in AMI compared to normal controls. 5 key DEmRNA biomarkers were identified based on machine learning, and classification diagnostic models were constructed. The random forests (RF) model has the highest accuracy. This indicates that RF model has high diagnostic value and may contribute to the early diagnosis of AMI. ROC analysis found that the area under curve of 5 key DEmRNA biomarkers were all greater than 0.7. Pearson correlation analysis showed that 5 key DEmRNA biomarkers were correlated with most of the differential infiltrating immune cells.

**Conclusion:**

The identification of new molecular biomarkers provides potential research directions for exploring the molecular mechanism of AMI. Furthermore, it is important to explore new diagnostic genetic biomarkers for the diagnosis and treatment of AMI.

**Supplementary Information:**

The online version contains supplementary material available at 10.1186/s12872-022-02999-7.

## Introduction

Acute myocardial infarction (AMI) is a necrotic event caused by an unstable ischemic syndrome with high morbidity and mortality. AMI can result in vascular disintegration and thinning of capillaries in the infarcted area [1]. Plaque rupture, plaque erosion and coronary microvascular dysfunction are common risk factors for myocardial infarction (MI) [2]. In addition to high mortality, AMI poses a severe economic burden. The early diagnosis and treatment of AMI can not only improve the therapeutic effect, but also reduce the economic pressure of patients [3]. Therefore, development of new AMI biomarkers is beneficial to improve diagnosis, treatment and management.

Diagnosis and prognostic detection of diseases at the molecular level are the current trend of medical development. The pathogenesis of AMI is complex, and its specific pathological mechanism remains unclear. However, several genes have been reported to be involved in the pathogenesis of AMI. DNA sequence variants in GATA binding protein 5 promoter can increase susceptibility to AMI [4]. Proprotein convertase subtilisin/kexin type 6 (PCSK6) play a key role in cardiac remodeling after acute myocardial infarction [5]. MiR-382-5p can affect apoptosis of myocardial cells after AMI by regulating stanniocalcin-1 (STC1) expression [6]. Overexpression of miR-147 can inhibit myocardial inflammation and apoptosis after MI and improve cardiac function by targeting homeodomain interacting protein kinase 2 (HIPK2) [7]. In addition, immunity also plays an important regulatory role in the progression of AMI. C-X-C motif chemokine receptor 4 affects tissue repair after MI by regulating regulatory T cells [8]. Activated B cells are involved in the sustained state of myocardial inflammation after AMI, and may affect the metabolism of myocardial collagen after AMI by secreting cytokines [9]. These studies suggest that studying the molecular mechanisms of AMI may be beneficial for the realization of new management approaches.

Machine learning is a growing field of medicine that integrates computer science and statistics into medical problems. It plays an important role in clinical diagnosis, precision treatment and health monitoring of diseases. Machine learning algorithms have been used to analyze the results of biomedical datasets, including random forests (RF), decision tree (DT), and support vector machine (SVM) [10]. Based on machine learning, clinical influencing factors of AMI were studied [11], the 1-year mortality of AMI was predicted [12], and a prediction model of arrhythmia after AMI was established [13]. Furthermore, a RF diagnostic model for AMI based on ferroptosis-related genes in circulating endothelial cells has been developed [14]. To date, machine learning has rarely been used to identify potential biomarkers of AMI. Therefore, in order to identify potential biomarkers of AMI, we performed machine learning algorithm analysis. In this study, all mRNA and miRNA data were downloaded from Gene Expression Omnibus (GEO) database. Subsequently, differentially expressed mRNAs (DEmRNAs) and differentially expressed miRNAs (DEmiRNAs) were identified and mRNA-mRNA network was constructed. Furthermore, 5 key DEmRNA biomarkers (ANPEP, REPS2, TUBB2A, ZNF281 and ARHGEF3) were identified based on machine learning and a classification diagnosis model was constructed. Our study has important value for understanding the pathological mechanism of AMI and exploring new diagnostic genetic biomarkers for the diagnosis and treatment of the disease.

## Materials and methods

### Source and processing of data

All mRNA and miRNA data were downloaded from the GEO database [15]. The keyword “acute myocardial infarction” and “homo sapiens” were searched in the GEO database. Datasets that meet the following criteria will be included in the study: (1) Dataset must be genome-wide mRNA or miRNA transcriptome data; (2) Data were obtained from blood samples of patients with AMI and normal controls; (3) Standardized or original datasets were considered. A total of 4 mRNA datasets (GSE66360, GSE48060, GSE34198 and GSE97320) and 1 miRNA dataset (GSE31568) were selected (Table 1). Download raw data from GSE66360, GSE48060, GSE34198 and GSE97320 and remove probes corresponding to multiple genes. For genes corresponding to multiple probes, only one probe with the highest average expression was retained. Scale normalization was performed on 17,044 mRNAs common to the 4 datasets. Subsequently, the datasets were merged and batch effects were removed with the ComBat function from the sva package.

**Table 1:** Details of the included dataset

GEO accession	Author	Platform	Samples (normal controls: AMI)	Year	Tissue	Data type
GSE66360	Eri Kramer	GPL570 [HG-U133_Plus_2] Affymetrix Human Genome U133 Plus 2.0 Array	50:49	2015	Blood	mRNA
GSE48060	XING LI	GPL570 [HG-U133_Plus_2] Affymetrix Human Genome U133 Plus 2.0 Array	21:31	2014	Blood	mRNA
GSE34198	Michal Kolář	GPL6102 Illumina human-6 v2.0 expression beadchip	48:49	2014	Blood	mRNA
GSE97320	Bo Fan Meng	GPL570 [HG-U133_Plus_2] Affymetrix Human Genome U133 Plus 2.0 Array	3:3	2017	Blood	mRNA
GSE31568	Andreas Keller	GPL9040 febit Homo Sapiens miRBase 13.0	70:20	2011	Blood	miRNA

### Differential expression analysis of mRNAs and miRNAs

DEmRNAs between AMI and normal controls were analyzed using metaMA package. The screening criterion was false discovery rate (FDR) < 0.05. DEmiRNAs between AMI and normal controls were analyzed using limma package. The screening criterion was FDR < 0.05.

### Functional enrichment analysis

To understand the biological functions of DEmRNAs, the Gene Ontology (GO) and Kyoto Encyclopedia of Genes and Genomes (KEGG) functional enrichment analysis of DEmRNAs was performed using genecodis database (https://genecodis.genyo.es/). GO enrichment analysis includes biological process (BP) terms, cell composition (CC) terms and molecular function (MF) terms. KEGG contains numerous signaling pathways [16–18]. The screening criterion was FDR < 0.05. In addition, “c2.cp.kegg.v7.4.symbols.gmt” was selected as the reference gene set, and GSEA 4.1.0 software was used for gene set enrichment analysis. The screening criterion was *P* < 0.05.

### Construction of miRNA-mRNA network

To explore the targeting relationship between DEmiRNAs and DEmRNAs, miRWalk (http://mirwalk.umm.uni-heidelberg.de/interactions/) was used to perform targeting relationship prediction for DEmiRNA. Relational pairs that were validated in at least one database (TargetScan, miRDB, MiRTarBase) were selected. Negative regulatory pairs involving DEmRNAs were used to construct the miRNA-mRNA network. Cytoscape (www.cytoscape.org/) was used to visualize the miRNA-mRNA network. Subsequently, GO and KEGG functional enrichment analysis was performed on target DEmRNAs in the miRNA-mRNA network. The screening criterion was FDR < 0.05.

### Identification of key DEmRNA biomarkers based on machine learning

RF (https://cran.r-project.org/web/packages/randomForest/) algorithm was used to construct classification model based on GSE66360 dataset. The importance of target DEmRNAs was sorted from large to small according to “Mean Decrease Accuracy” value. According to the sorting order, add one DEmRNAs from top to bottom. Then, RF algorithm was used for classification, and ten-fold cross-validation was used to obtain accuracy and area under curve (AUC). Then, key DEmRNA biomarkers were selected to construct SVM, RF, and DT classification models. The accuracy, sensitivity, specificity and AUC values in the receiver operating characteristic (ROC) curve of the three classification models obtained by the tenfold cross-validation were used to evaluate the potential diagnostic ability of classification model. To further verify the potential diagnostic value of the classification models constructed by the key DEmRNA biomarkers, we performed hold-out validation. The GSE66360 data was randomly divided into two datasets, test1 (Control:AMI = 25:25) and test2 (Control:AMI = 25:24). The accuracy, sensitivity, specificity and AUC values in the ROC curves of the three classification models were obtained by hold-out validation. In addition, classification models were also constructed using key DEmRNA biomarkers based on the total data (GSE66360, GSE48060, GSE34198 and GSE97320). The ten-fold cross-validation was used to obtain AUC.

### Diagnostic and expression of key DEmRNA biomarkers

To further investigate the potential diagnostic value of key DEmRNA biomarkers, we performed diagnostic analyses using GSE66360 dataset. The ROC analysis was performed using pROC package in R language to predict the diagnostic accuracy of key DEmRNA biomarkers. The sensitivity and specificity at the cut-offs were determined according to a previous study [19]. In ROC analysis, the greater the AUC, the higher the diagnostic accuracy [20]. AUC > 0.6 indicates sufficient diagnostic accuracy. Boxplots of key DEmRNA biomarkers in AMI and normal controls were also drawn. In addition, the expression of key DEmRNA biomarkers was also validated on the GSE60993 dataset.

### Immune correlation analysis

The CIBERSORT package was used to perform analysis of immune cell infiltration level according to gene expression matrix. Statistical analysis of immune cell infiltration levels was performed using the rank-sum test. To further understand the potential role of key DEmRNA biomarkers, the correlation between immune cells and key DEmRNA biomarkers was analyzed using Pearson correlation coefficient.

### Statistical analysis

All statistics were performed using R software. The metaMA and limma packages are used to analyze DEmRNAs and DEmiRNAs, respectively. The screening criterion was FDR < 0.05. Rank-sum test was used to analyze the statistical difference of immune cell infiltration level between AMI and normal control groups. *P* < 0.05 was considered to be statistically significant.

## Results

### Identification of DEmRNAs and DEmiRNAs

According to the screening criteria FDR < 0.05, 92 DEmRNAs that were consistently up-regulated and down-regulated in the 4 datasets were screened (Additional file 1: Fig. 1A). Among them, 80 were up-regulated and 12 were down-regulated. Top 10 up-regulated DEmRNAs were CDA, MMP9, LRG1, MXD1, C19orf59, LY96, ADM, ANPEP, PLBD1 and SMAP2 (Additional file 4: Table S1). Inversely, top 10 down-regulated DEmRNAs were KLRG1, PSMA5, VPS29, CD38, ANAPC5, LIMS1, ARHGEF3, HINT1, RPS24 and GBP5 (Additional file 4: Table S1). At the same time, 272 DEmiRNAs were identified (Additional file 1: Fig. 1B). Among them, 124 were up-regulated and 148 were down-regulated. Top 10 up-regulated DEmiRNAs were hsa-miR-1290, hsa-miR-302b, hsa-miR-126*, hsa-miR-302d, hsa-miR-1468, hsa-miR-1258, hsa-miR-508-3p, hsa-miR-609, hsa-miR-27a and hsa-miR-892b (Additional file 5: Table S2). Inversely, top 10 down-regulated DEmiRNAs were hsa-miR-1283, hsa-miR-31*, hsa-miR-518a-3p, hsa-miR-519e*, hsa-miR-488*, hsa-miR-566, hsa-miR-1278, hsa-miR-1291, hsa-miR-515-5p, hsa-miR-591 (Additional file 5: Table S2).

### Functional enrichment analysis of DEmRNAs

**Fig. 1:**
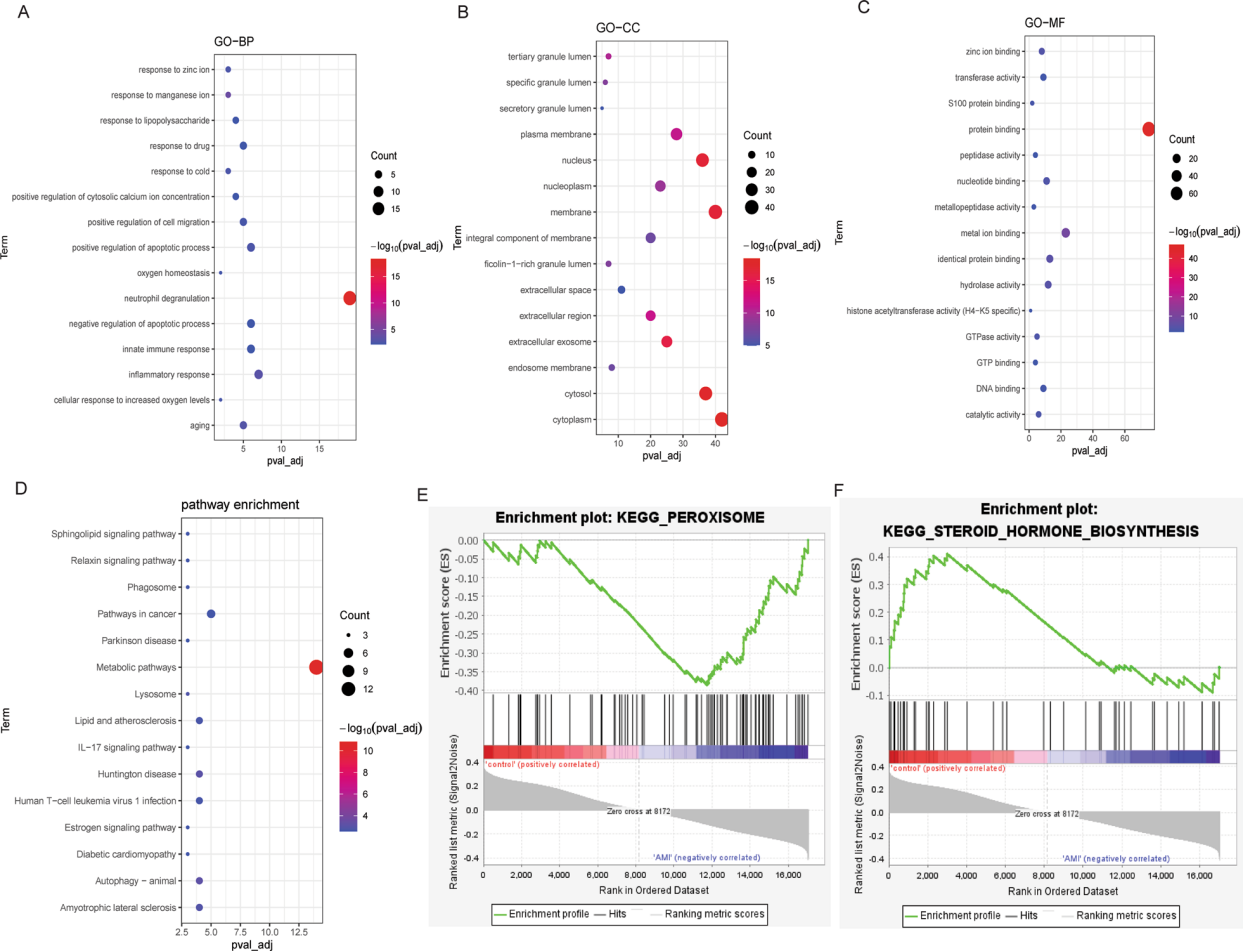
Functional enrichment analysis of DEmRNAs. **A** Top 15 significantly enriched Gene Ontology (GO): biological process (BP) terms; **B** Top 15 significantly enriched GO: cell composition (CC) terms; **C** Top 15 significantly enriched GO: molecular function (MF) terms; **D** Top 15 signaling pathways of Kyoto Encyclopedia of Genes and Genomes (KEGG) enrichment analysis. **E** Gene Set Enrichment Analysis (GSEA) analysis showed that steroid hormone biosynthesis signaling pathway was down-regulated in acute myocardial infarction (AMI); **F** GSEA analysis showed that peroxisome signaling pathway was down-regulated in AMI

In order to explore the biological functions of DEmRNAs, functional enrichment analysis was performed. In GO: BP, DEmRNAs were mainly involved in neutrophil degranulation, signal transduction and inflammatory response (Fig. 1A). In GO: CC, DEmRNAs were mainly distributed in the cytoplasm, membrane and cytosol (Fig. 1B). In GO: MF, DEmRNAs were mainly associated with protein binding, metal ion binding and identical protein binding (Fig. 1C). KEGG analysis showed that DEmRNAs was significantly enriched in metabolic pathways, pathways in cancer and amyotrophic lateral sclerosis (Fig. 1D). Furthermore, GSEA analysis found that gene set kegg_peroxisome (*P* = 0.037) was up-regulated in AMI (Fig. 1E) and gene set kegg_steroid_hormone_biosynthesis (*P* = 0.047) was down-regulated in AMI (Fig. 1F) compared to normal controls.

### Construction of miRNA-mRNA network and functional enrichment of target DEmRNAs

**Fig. 2:**
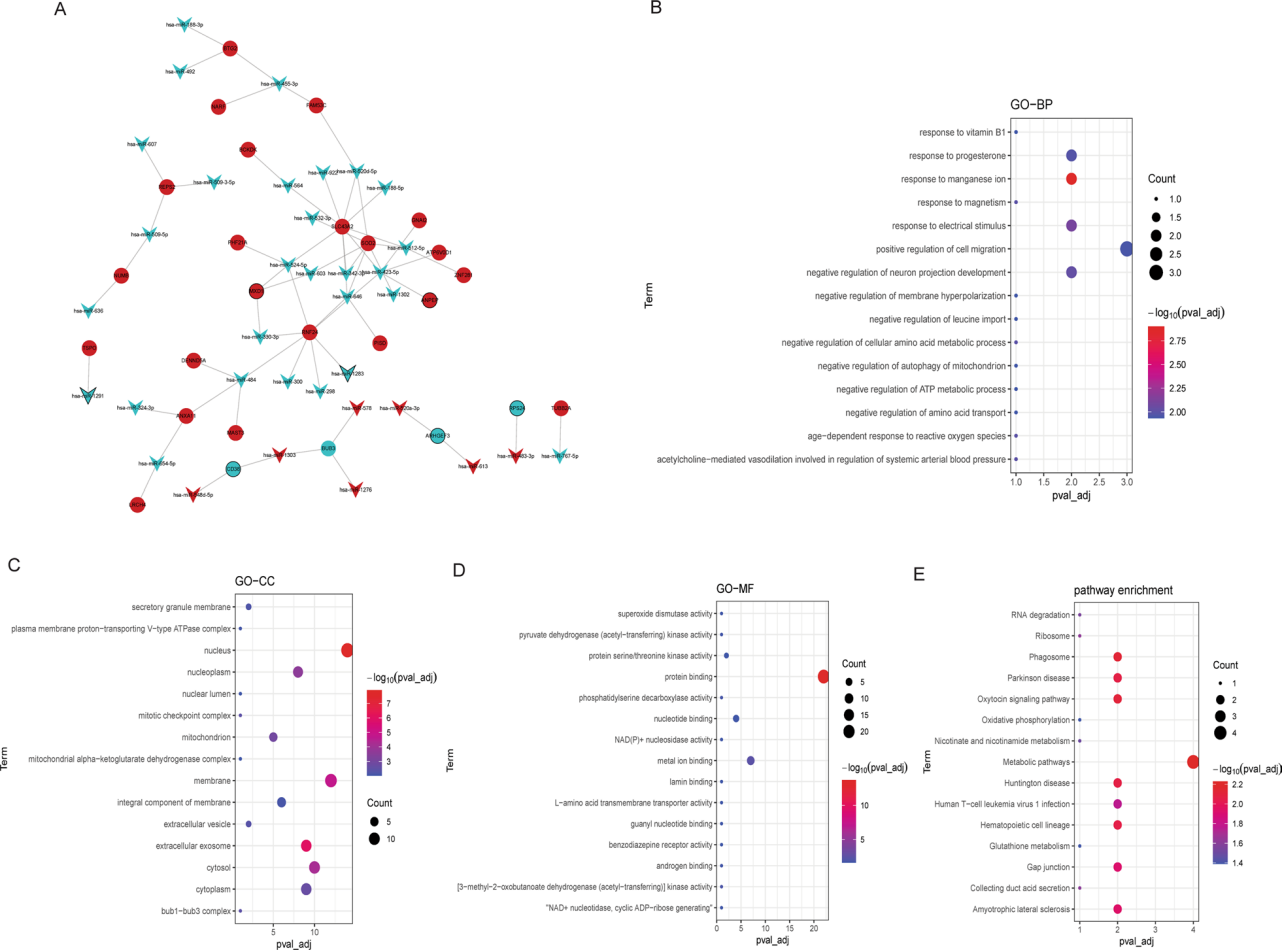
Construction of miRNA-mRNA network and functional enrichment of target DEmRNAs. **A** MiRNAs-mRNA network. V-shape, circle, blue and red represent miRNA, mRNA, down-regulation, and up-regulation, respectively. The thick black borders are the up-regulated or down-regulated DEmiRNAs or DEmRNAs in top10. **B** Top 15 significantly enriched Gene Ontology (GO): biological process (BP) terms; **C** Top 15 significantly enriched GO: cell composition (CC) terms; **D** Top 15 significantly enriched GO: molecular function (MF) terms **E** Top 15 signaling pathways of Kyoto Encyclopedia of Genes and Genomes (KEGG) enrichment analysis

MiRWalk was used to predict the target mRNAs of 272 DEmiRNAs. Negative regulatory pairs involving DEmRNAs were used to construct the miRNA-mRNA network. Cytoscape was used to visualize the miRNA-mRNA network. A total of 132 negatively regulated targeting relationship pairs were obtained in the miRNA-mRNA network. In addition, the 132 negative regulatory targeting relationship pairs include 26 DEmRNAs and 35 DEmiRNAs (Fig. 2A). GO and KEGG functional enrichment analysis of 26 target DEmRNAs was performed using the genecodis database. In GO: BP, target DEmRNAs were mainly involved in positive regulation of cell migration and response to manganese ion (Fig. 2B). In GO: CC, target DEmRNAs were mainly distributed in the nucleus and membrane (Fig. 2C). In GO: MF, target DEmRNAs were mainly associated with protein binding and metal ion binding (Fig. 2D). KEGG analysis showed that target DEmRNAs was significantly enriched in metabolic pathways, phagosome and oxytocin signaling pathway (Fig. 2E).

### Identification of key DEmRNA biomarkers and construction of classification models

**Fig. 3:**
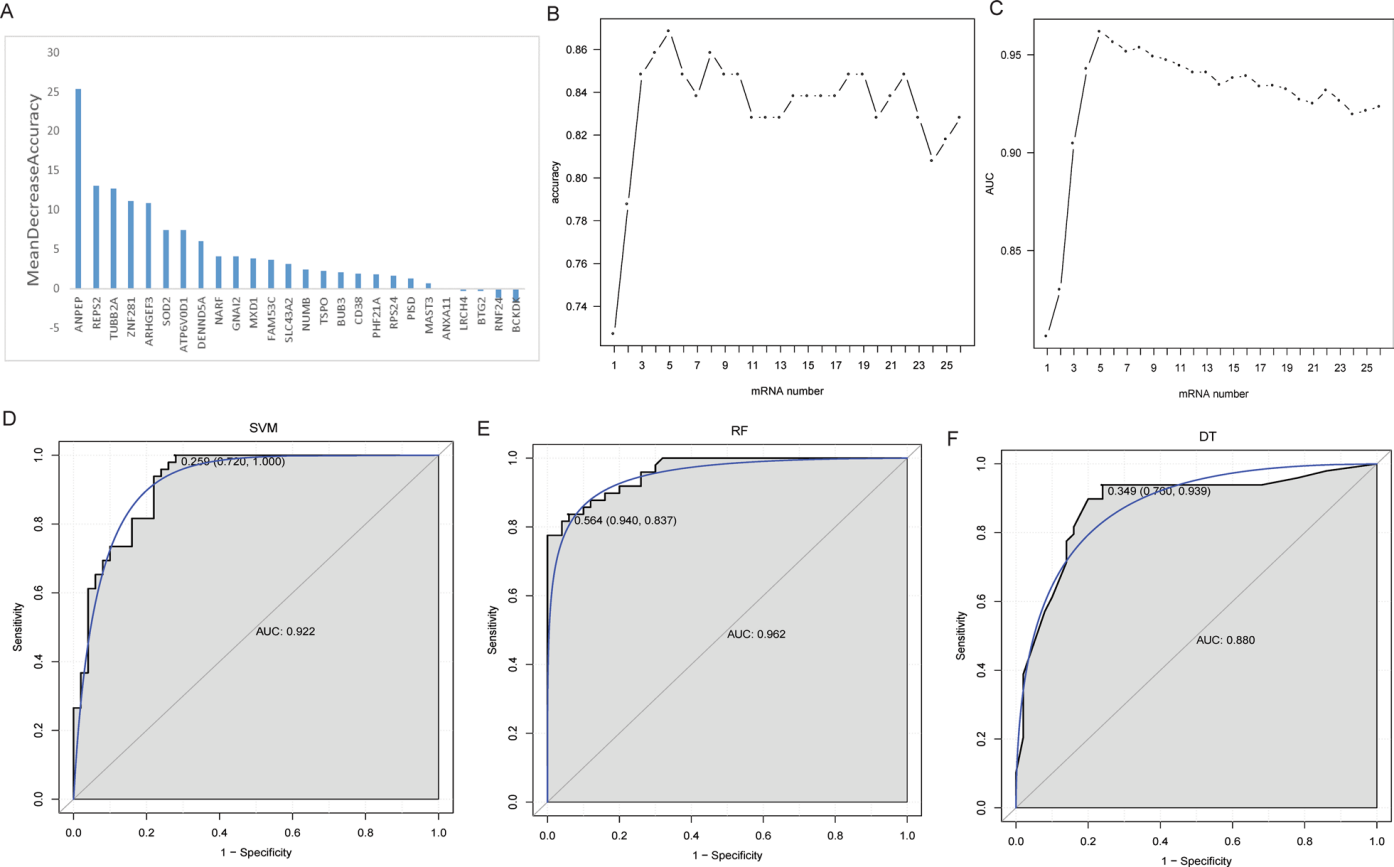
Identification of key DEmRNA biomarkers and construction of classification models. **A** The importance ranking map of 26 target DEmRNAs; **B** Trend chart of accuracy with the increase of DEmRNA quantity; **C** Trend chart of area under curve (AUC) with the increase of DEmRNA quantity; **D** Receiver operating characteristic (ROC) curves of the support vector machine (SVM) classifier; **E** ROC curves of the random forests (RF) classifier; **F** ROC curves of the decision tree (DT) classifier

Based on “Mean Decrease Accuracy” value, the importance of 26 target DEmRNAs was sorted from large to small (Fig. 3A). According to the sorting order, add one DEmRNAs from top to bottom. Then, RF algorithm was used for classification, and ten-fold cross-validation was used to obtain accuracy and AUC. The results showed that accuracy and AUC value reached the maximum value when the number of DEmRNA reached 5 (Fig. 3B and C). Therefore, these 5 DEmRNAs (ANPEP, REPS2, TUBB2A, ZNF281 and ARHGEF3) were selected as key biomarkers. SVM, RF and DT classification models were constructed based on 5 key DEmRNA biomarkers. The results show that the RF model has the highest accuracy (0.859) (Table 2). The AUC in the ROC curve of SVM, RF and DT were 0.922, 0.962, and 0.880, respectively (Fig. 3D–F). To further verify the potential diagnostic value of the classification models constructed by the 5 key DEmRNA biomarkers, we performed hold-out validation. The results show that the accuracy of the RF classification model for test1 and test2 datasets was greater than 0.8 (Additional file 6: Table S3). In test1 data, the AUC of SVM, RF and DT were 0.862, 0.934 and 0.863, respectively (Additional file 2: Fig. 2A–C). In test2 data, the AUC of SVM, RF and DT were 0.930, 0.958 and 0.763, respectively (Additional file 2: Fig. 2D–F). In addition, three classification models were also constructed using 5 key DEmRNA biomarkers based on the total data (GSE66360, GSE48060, GSE34198 and GSE97320). The tenfold cross-validation was used to obtain AUC. The AUC of SVM, RF and DT were 0.719, 0.706, and 0.603, respectively (Additional file 2: Fig. 2G–I). These results imply that the classification models based on 5 key DEmRNA biomarkers have sufficient diagnostic accuracy.

**Table 2:** Tenfold cross-validation results of each model

Classifier	Accuracy	Sensitivity	Specificity	Area under curve (AUC)
Support vector machine (SVM)	0.818	0.796	0.840	0.922
Random forest (RF)	0.859	0.898	0.820	0.962
Decision tree (DT)	0.818	0.776	0.860	0.880

### Diagnostic and expression of key DEmRNA biomarkers

**Fig. 4:**
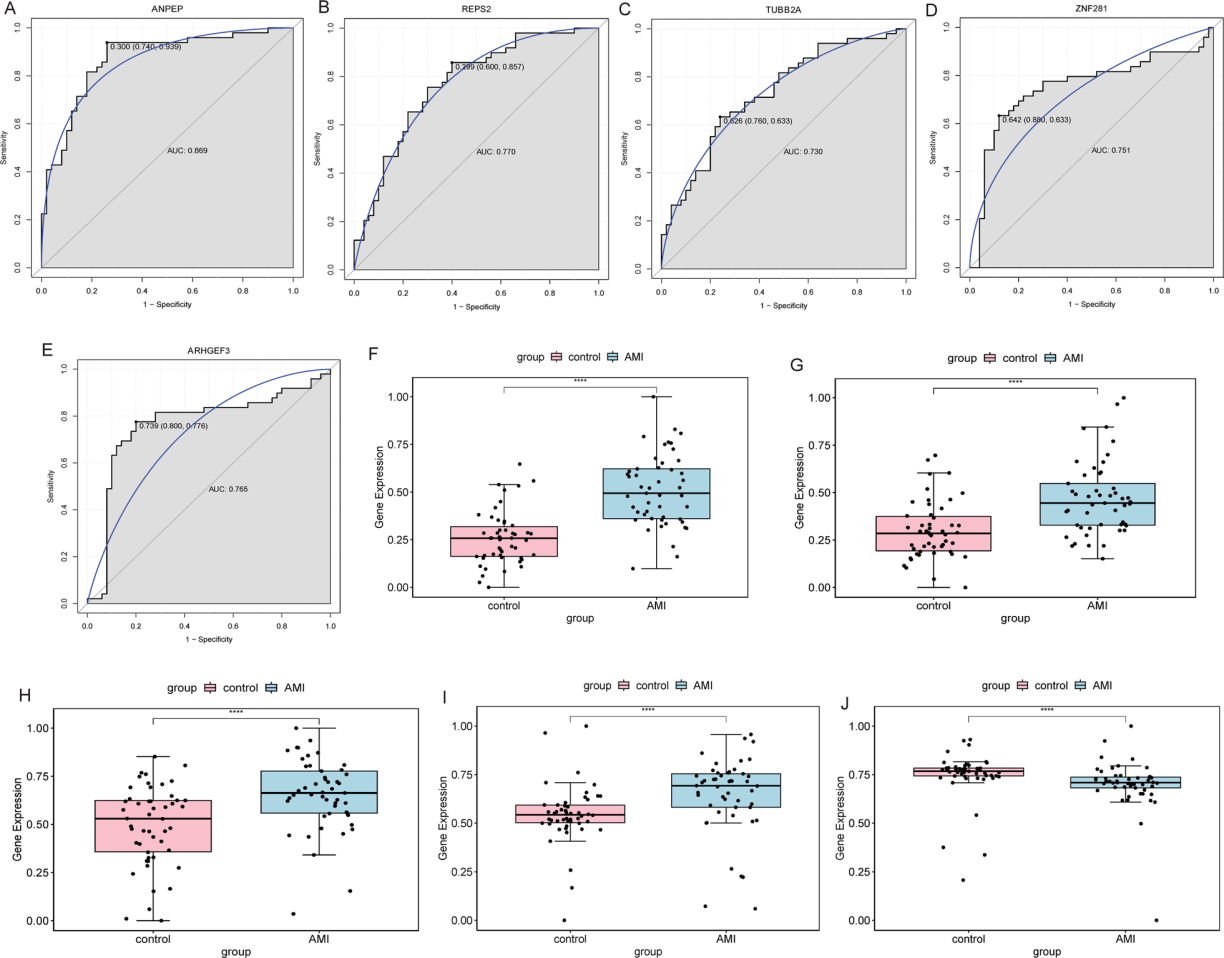
Receiver operating characteristic (ROC) diagnostic analysis and expression analysis of key DEmRNA biomarkers in GSE66360 dataset. **A** ROC diagnostic analysis of the ANPEP; **B** ROC diagnostic analysis of the REPS2; **C** ROC diagnostic analysis of the TUBB2A; **D** ROC diagnostic analysis of the ZNF281; **E** ROC diagnostic analysis of the ARHGEF3; **F** Expression analysis of ANPEP; **G** Expression analysis of REPS2; **H** Expression analysis of TUBB2A; **I** Expression analysis of ZNF281; **J** Expression analysis of ARHGEF3

**Fig. 5:**
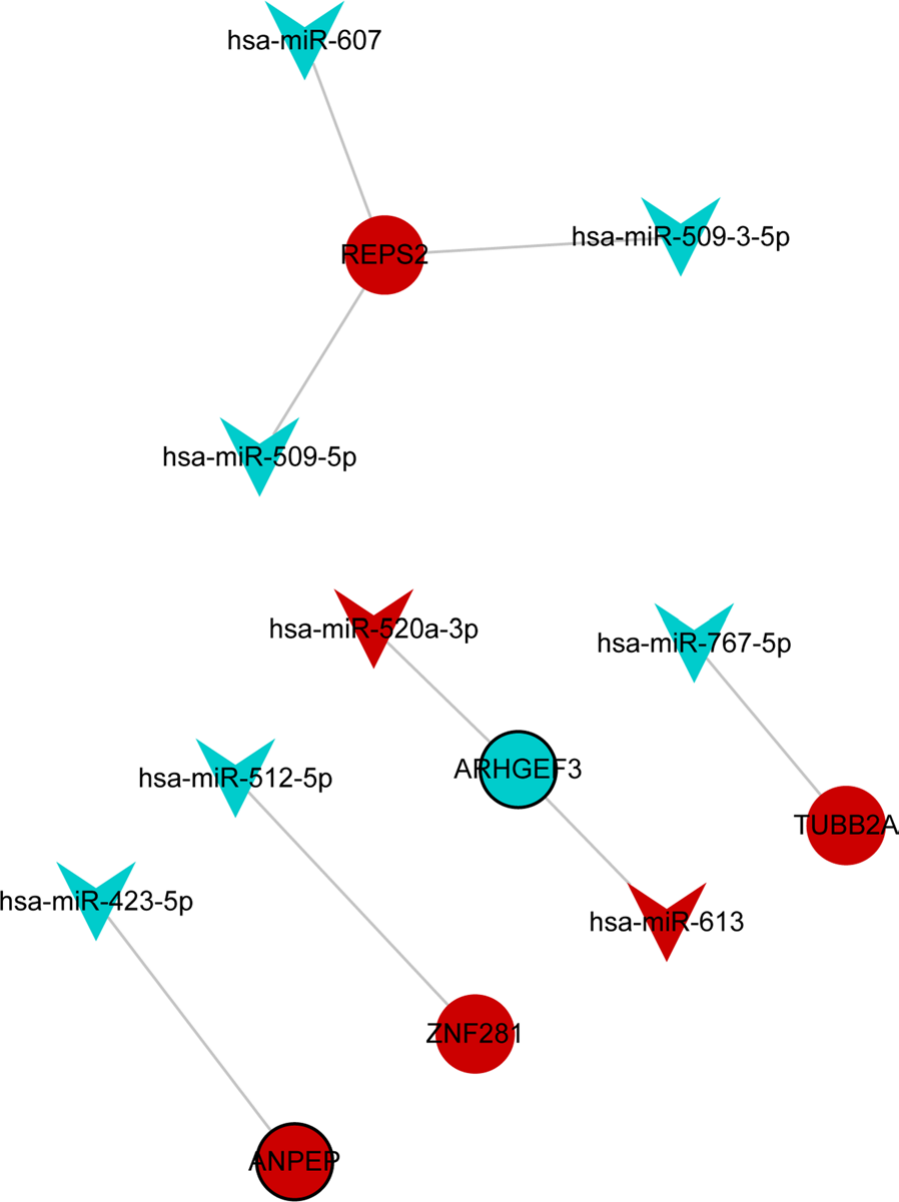
MiRNA-key DEmRNA biomarkers sub-network. V-shape, circle, blue and red represent miRNA, mRNA, down-regulation, and up-regulation, respectively. The thick black borders are the up-regulated or down-regulated DEmiRNAs or DEmRNAs in top10

ROC analysis was performed for 5 key DEmRNA biomarkers to evaluate their diagnostic value. The AUC of 5 key DEmRNA biomarkers were all greater than 0.7 (Fig. 4A–E). The result showed that these 5 key DEmRNA biomarkers may be considered as the potential diagnostic biomarkers in AMI. However, in GSE66360 dataset, compared with SVM, RF and DT classification models, the AUC of 5 key DEmRNA biomarkers was lower than that of all models. This further implies that classification models may have higher potential diagnostic value compared with individual key biomarkers. Furthermore, ANPEP, REPS2, TUBB2A and ZNF281 were up-regulated in AMI (Fig. 4F–I) and ARHGEF3 was down-regulated (Fig. 4J) in AMI compared with normal controls. Subsequently, mRNA-miRNA sub-networks containing only key DEmRNA biomarkers were screened from the mRNA-miRNA network. Include 5 key DEmRNA biomarkers and 8 DEmiRNAs in the sub-network (Fig. 5). Subsequently, the expression of 5 key DEmRNA biomarkers was verified in the GSE60993 dataset. The results showed that the expression trend of 5 key DEmRNA biomarkers was consistent with that in the GSE66360 dataset (Additional file 3: Fig. 3).

### Correlation analysis between immune cells and key DEmRNA biomarkers

**Fig. 6:**
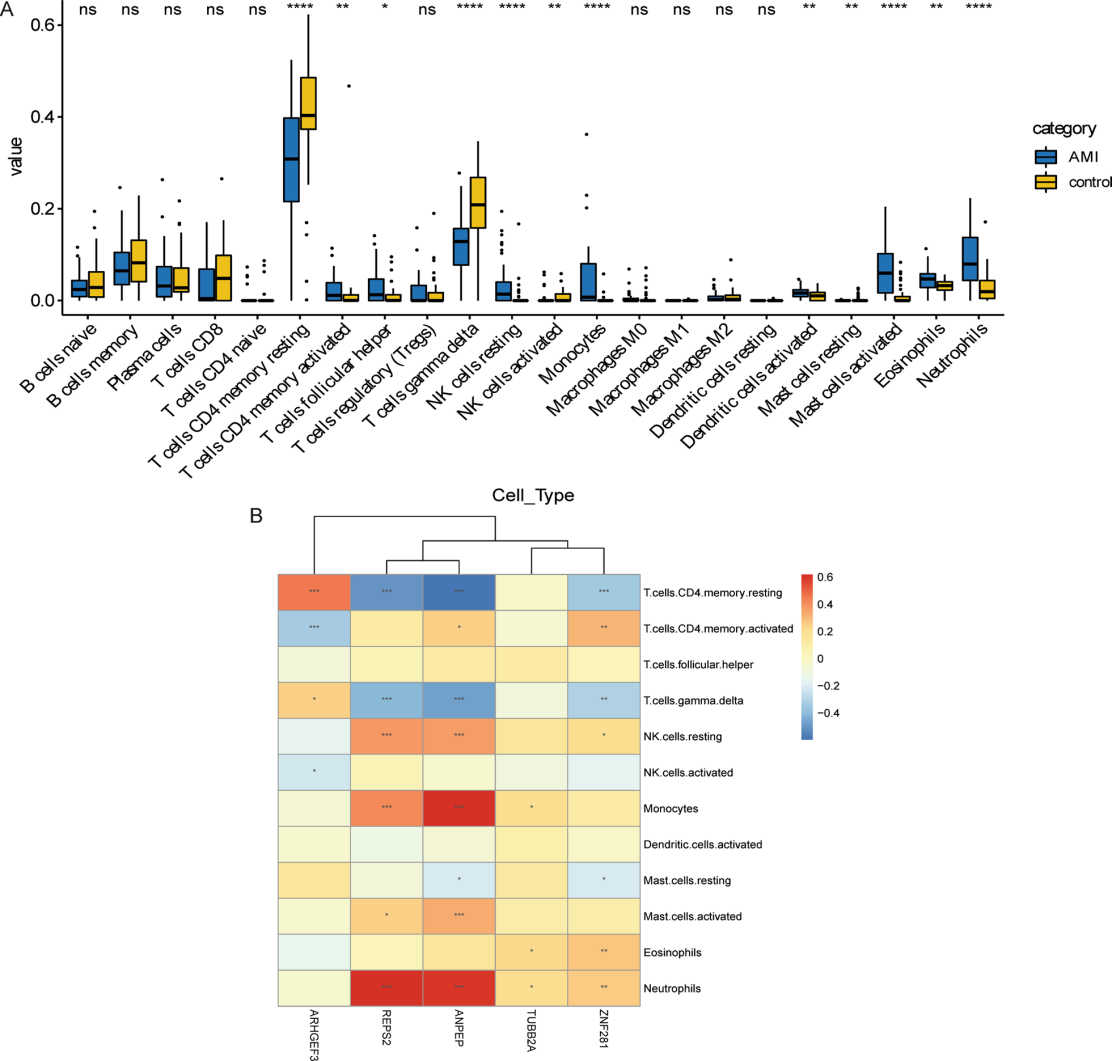
Immune correlation analyses. **A** Distribution of immune cells between normal control and AMI groups; **B** Pearson correlation coefficient analysis the correlation between immune cells and 5 key DEmRNA biomarkers. The darker the color, the stronger the correlation. Red and blue represent positive and negative correlations, respectively. * represent *P* < 0.05, ** represent *P* < 0.01, *** represent *P* < 0.001, **** represent *P* < 0.0001; ns represent no statistical significance

The distribution of 12 immune cell types was significantly different between normal controls and AMI by rank-sum test (Fig. 6A). Pearson correlation analysis showed that 5 key DEmRNA biomarkers were correlated with most of the differential infiltrating immune cells (Fig. 6B). The results demonstrated that REPS2 was significantly positively correlated with neutrophils. Moreover, ANPEP was significantly positively correlated with neutrophils and monocytes, and negatively correlated with T cells CD4 memory resting. This implies that key biomarkers play important roles in the immune regulation of AMI.

## Discussion

AMI is one of the main diseases threatening human life. Early diagnosis and treatment can reduce mortality and improve the prognosis of AMI. In order to identify potentially effective biomarkers, we analyzed AMI data based on GEO database. In this study, 92 DEmRNAs and 272 DEmiRNAs were identified. Functional analysis showed that DEmRNAs were enriched in various biological processes, suggesting the complexity of AMI pathogenesis. Then, 5 key DEmRNA biomarkers (ANPEP, REPS2, TUBB2A, ZNF281 and ARHGEF3) were obtained by RF analysis of 26 target DEmRNAs based on the scale data of 17,044 mRNAs, and classification diagnosis models of RF, DT and SVM were constructed. The AUC in the ROC curve of SVM, RF and DT were 0.922, 0.962, and 0.880, respectively. Moreover, the RF model has the highest accuracy (0.859). These results suggest that RF model has high diagnostic value and may contribute to the early diagnosis of AMI. To further investigate the potential diagnostic value of 5 key DEmRNA biomarkers, we performed ROC analyses. The AUC of 5 key DEmRNA biomarkers were all greater than 0.7. The result showed that 5 key DEmRNA biomarkers may be considered as potential diagnostic biomarkers in AMI. However, compared with SVM, RF and DT classification models, the AUC of 5 key DEmRNA biomarkers was lower than that of all models. This further implies that classification models may have higher potential diagnostic value compared with individual key biomarkers. In addition, we also found 5 key biomarkers associated with immune cells. This implies that key biomarkers play important roles in the immune regulation of AMI.

Alanyl aminopeptidase, membrane (ANPEP, also known as CD13 and APN) is essential for inflammatory transport and infarct healing after permanent coronary artery occlusion [21]. ANPEP can regulate the repair of atherosclerotic vascular injury [22]. The disorder of ANPEP is also related to the pathogenesis of hypertension [23]. To our knowledge, this is the first study to show that ANPEP is differentially expressed in AMI and may be a potential diagnostic biomarker for AMI. KEGG analysis found that metabolic pathways, glutathione metabolism, hematopoietic cell lineage, and renin-angiotensin system were significantly enriched signaling pathways (FDR < 0.05), and involved multiple DEmRNAs including ANPEP. Patients with AMI have abnormal metabolic pathways [24, 25]. Abnormal glutathione levels in AMI are associated with oxidative stress mechanisms [26]. Hematopoietic stem cells have potential regenerative capacity in AMI xenotransplantation [27]. Wang S et al. also found that hematopoietic cell lineage has potential regulatory roles in AMI [28]. Renin-angiotensin system plays an important role in the mediation of AMI [29]. Exosomes prevent AMI in rats by modulating the renin-angiotensin system [30]. Therefore, we speculate that ANPEP may play an important role in the ongoing case mechanism of AMI by regulating metabolic pathways, glutathione metabolism, hematopoietic cell lineage, and renin-angiotensin system with other mRNAs. In addition, we also predicted that ANPEP (up-regulated) and hsa-miR-423-5p (down-regulated) have negative targeting relationship based on the miRWalk database. Previous studies have found that hsa-miR-423-5p is abnormally expressed in AMI and is associated with the mortality of cardiogenic shock [31–33]. Based on this study, we speculated that ANPEP may be a potential target gene for hsa-miR-423-5p to play a regulatory role in AMI. Remarkably, we also found that ANPEP was significantly associated with neutrophils, monocytes and T cells CD4 memory resting. Neutrophils [34], monocytes [35] and T cells CD4 memory resting [36] play important roles in the pathophysiology of AMI. This further suggests that ANPEP may mediate the progression of AMI by regulating immune cells.

Tubulin beta 2A class IIa (TUBB2A) protein is a major component of microtubule structure [37]. Previous studies have found that TUBB2A is up-regulated in acute myocardial infarction, which is consistent with our results [38]. KEGG analysis found that Parkinson disease, gap junction and phagosomes were significantly enriched signaling pathways (FDR < 0.05), and involved multiple DEmRNAs including TUBB2A. Studies have found that Parkinson’s patients are more likely to suffer from AMI [39]. Enhancement of gap junction function during AMI improves healing and reduces susceptibility to late ventricular arrhythmias [40]. Phagosomes are involved in tissue remodeling, clearing apoptotic cells and limiting the spread of intracellular pathogens [41]. Phagosomes are closely related to autophagy [42]. Autophagosomes are present in surviving cardiomyocytes in the chronic phase of MI to inhibit apoptosis and mitigate harmful effects, which may alleviate cardiac dysfunction and enhance remodeling [43]. Therefore, we speculate that TUBB2A may play an important role in the ongoing case mechanism of AMI by regulating Parkinson disease, gap junction and phagosomes with other mRNAs. In addition, we also predicted that TUBB2A (up-regulated) and hsa-miR-767-5p (down-regulated) have negative targeting relationship based on the miRWalk database. However, there is no evidence for the role of miR-767-5p in AMI. These results provide a direction for further exploring the molecular mechanism of TUBB2A in AMI.

Previous studies on RALBP1 associated Eps domain containing 2 (REPS2, also known as POB1) mostly focus on cancer, such as breast cancer [44], esophageal squamous cell carcinoma [45] and prostate cancer [46]. Transcription Factor zinc finger protein 281 (ZNF281) play a regulatory role in intestinal fibrosis [47]. ZNF281 acts at a nexus of cardiac and inflammatory gene programs, which exert influences on fibroblast and cardiac reprogramming [48]. In this study, rho guanine nucleotide exchange factor 3 (ARHGEF3, also known as XPLN) was the only down-regulated DEmRNA biomarker among 5 key DEmRNA biomarkers. A study has shown that ARHGEF3 plays a regulatory role in pulmonary fibrosis through mTORC2 [49]. Modulation of ARHGEF3 gene expression plays a role in human megakaryocytes and platelet function [50]. To our knowledge, this is the first study to show a potential regulatory role of REPS2, ZNF281 and ARHGEF3 in AMI progression. This provides a new theoretical basis for studying the molecular mechanism of AMI. In addition, we also predicted that REPS2 (up-regulated) and hsa-miR-509–3-5p (down-regulated), hsa-miR-509-5p (down-regulated) and hsa-miR-607 (down-regulated) have negative targeting relationships, ZNF281 (up-regulated) and hsa-miR-512-5p (down-regulated) have negative targeting relationships, and ARHGEF3 (down-regulated) and hsa-miR-520a-3p (up-regulated) and hsa-miR-613 (up-regulated) have negative targeting relationships based on the miRWalk database. These results indicate that REPS2, ZNF281 and ARHGEF3 are regulated by other molecules in AMI. Remarkably, REPS2 was significantly positively correlated with neutrophils. This suggests that REPS2 may play a role in the dysregulation of immune regulation in AMI. The identification of REPS2, ZNF281 and ARHGEF3 and related miRNAs provides potential research directions for further exploring the molecular mechanism of AMI.

GSEA analysis found that peroxisome was up-regulated in AMI and steroid hormone biosynthesis was down-regulated in AMI compared to normal controls. The peroxisome proliferator-activated receptor-gamma plays a role in promoting cardiac healing after AMI [51]. Peroxisome proliferator-activated receptor down-regulates the expression of pro-inflammatory molecules in MI [52]. Sex hormone is an important regulator of acute inflammatory response after cardiac injury [53]. Hormone therapy with estrogen can reduce the risk of AMI [54]. Therefore, further exploration of the molecular mechanisms of peroxisome and steroid hormone biosynthesis signaling pathways are conducive to understanding the molecular regulatory mechanism of AMI.

However, this study has a certain degree of limitations. All data in this study came from public databases, lacking clinical sample validation. Therefore, clinical samples need to be collected for further research at a later stage. Furthermore, the specific roles of the identified key mRNA biomarkers and related biological pathways and miRNAs in AMI remain unclear. Therefore, further studies in vitro are required to understand the molecular mechanism of AMI.

In summary, 5 key DEmRNA biomarkers were obtained by RF analysis of 26 target DEmRNAs based on the scale data of 17,044 mRNAs, and classification diagnosis models of RF, DT and SVM were constructed. The AUC in the ROC curve of SVM, RF and DT were 0.922, 0.962, and 0.880, respectively. Moreover, the RF model also has the highest accuracy. These results indicate that the RF model has high diagnostic value and may contribute to the early diagnosis of AMI. In addition, we found 5 key biomarkers associated with immune cells. Identification of new molecular biomarkers provides potential research directions for exploring the molecular mechanism of AMI. Furthermore, it is important to explore new diagnostic genetic biomarkers for the diagnosis and treatment of AMI.

## Supplementary Information

**Additional file 1: Fig. S1 **Volcano plot of DEmRNAs **A** and Volcano plot of DEmiRNAs **B**. DEmRNAs, differentially expressed mRNAs; DEmiRNAs, differentially expressed miRNAs.

**Additional file 2: Fig. S2 **Validation of classification models constructed by 5 key DEmRNA biomarkers. **A**: Receiver operating characteristic (ROC) curves of the support vector machine (SVM) classifier in test1; **B**: ROC curves of the random forests (RF) classifier in test1; **C**: ROC curves of the decision tree (DT) classifier in test1; **D**: ROC curves of the SVM classifier in test2; **E**: ROC curves of the RF classifier in test2; **F**: ROC curves of the DT classifier in test2; **G**: ROC curves of the SVM classifier in total data (GSE66360, GSE48060, GSE34198 and GSE97320); **H**: ROC curves of the RF classifier in total data; **I**: ROC curves of the DT classifier in total data.

**Additional file 3: Fig. S3 **Expression analysis of key DEmRNA biomarkers in GSE60993 dataset. **A**: Expression analysis of ANPEP; **B**: Expression analysis of REPS2; **C**: Expression analysis of TUBB2A; **D**: Expression analysis of ZNF281; **E**: Expression analysis of ARHGEF3. * represent *P* < 0.05, ** represent *P* < 0.01; ns represent no statistical significance.

**Additional file 4: Table S1 **Top 10 up-regulated and down-regulated DEmRNAs.

**Additional file 5: Table S2 **Top 10 up-regulated and down-regulated DEmiRNAs.

**Additional file 6: Table S3 **hold-out validation results of each model.

## Data Availability

The database analysed during the current study are available in the GEO database, persistent accessible web link to database is https://www.ncbi.nlm.nih.gov/geo/. Accession numbers of the datasets used in the current study are GSE66360, GSE48060, GSE34198, GSE97320 and GSE31568 in Gene Expression Omnibus (https://www.ncbi.nlm.nih.gov/geo). All data generated or analyzed during this study are included in this published article.
