# Supplementary material for: Uncovering potential diagnostic biomarkers of acute myocardial infarction based on machine learning and analyzing its relationship with immune cells
Source: BMC Cardiovasc Disord. 2023 Jan 4;23:2. doi: 10.1186/s12872-022-02999-7 (PMC9814319; doi:10.1186/s12872-022-02999-7)
Supplement: Supplementary file 4 — Additional file 4: Table S1 Top 10 up-regulated and down-regulated DEmRNAs. [file 12872_2022_2999_MOESM4_ESM.docx]

**Table S1 Top 10 up-regulated and down-regulated DEmRNAs**

| **ID** | **Symbol** | **Combined.ES** | **P.Value** | **False discovery rate (FDR)** | **Up/Down** |
| --- | --- | --- | --- | --- | --- |
| 978 | CDA | 0.923914553 | 1.37E-10 | 2.47E-10 | Up |
| 4318 | MMP9 | 0.842386401 | 5.44E-10 | 9.79E-10 | Up |
| 116844 | LRG1 | 0.848782647 | 7.00E-10 | 1.26E-09 | Up |
| 4084 | MXD1 | 0.788122304 | 2.85E-09 | 5.14E-09 | Up |
| 199675 | C19orf59 | 0.758623369 | 1.76E-08 | 3.17E-08 | Up |
| 23643 | LY96 | 0.744396939 | 5.59E-08 | 1.00E-07 | Up |
| 133 | ADM | 0.737365577 | 7.59E-08 | 1.36E-07 | Up |
| 290 | ANPEP | 0.730485591 | 1.49E-07 | 2.67E-07 | Up |
| 79887 | PLBD1 | 0.689278768 | 1.23E-06 | 2.19E-06 | Up |
| 64744 | SMAP2 | 0.615656444 | 4.05E-06 | 7.15E-06 | Up |
| 10219 | KLRG1 | -0.662185405 | 7.65E-07 | 1.37E-06 | Down |
| 5686 | PSMA5 | -0.411759926 | 0.001153879 | 0.001844886 | Down |
| 51699 | VPS29 | -0.341749425 | 0.007044443 | 0.010680749 | Down |
| 952 | CD38 | -0.338923291 | 0.007411018 | 0.011208622 | Down |
| 51433 | ANAPC5 | -0.3227364 | 0.010993958 | 0.016420587 | Down |
| 3987 | LIMS1 | -0.316717574 | 0.013919334 | 0.020614648 | Down |
| 50650 | ARHGEF3 | -0.308463564 | 0.014238341 | 0.021059643 | Down |
| 3094 | HINT1 | -0.310846178 | 0.014334412 | 0.021187026 | Down |
| 6229 | RPS24 | -0.306709982 | 0.015010438 | 0.02215356 | Down |
| 115362 | GBP5 | -0.287573953 | 0.02320802 | 0.0337091 | Down |
