# Supplementary material for: Uncovering potential diagnostic biomarkers of acute myocardial infarction based on machine learning and analyzing its relationship with immune cells
Source: BMC Cardiovasc Disord. 2023 Jan 4;23:2. doi: 10.1186/s12872-022-02999-7 (PMC9814319; doi:10.1186/s12872-022-02999-7)
Supplement: Supplementary file 5 — Additional file 5: Table S2 Top 10 up-regulated and down-regulated DEmiRNAs. [file 12872_2022_2999_MOESM5_ESM.docx]

**Table S2 Top 10 up-regulated and down-regulated DEmiRNAs**

| **Symbol** | **logFC (log Fold Change)** | **P.Value** | **adj.P.Val (false discovery rate)** | **Up/Down** |
| --- | --- | --- | --- | --- |
| hsa-miR-1290 | 1.017687 | 8.39E-11 | 3.62E-08 | Up |
| hsa-miR-302b | 1.081274 | 1.68E-09 | 4.83E-07 | Up |
| hsa-miR-126* | 0.974867 | 6.30E-09 | 9.07E-07 | Up |
| hsa-miR-302d | 0.81684 | 5.59E-07 | 2.75E-05 | Up |
| hsa-miR-1468 | 0.827398 | 8.03E-07 | 3.46E-05 | Up |
| hsa-miR-1258 | 0.900551 | 9.89E-07 | 3.88E-05 | Up |
| hsa-miR-508-3p | 0.945046 | 1.13E-06 | 4.07E-05 | Up |
| hsa-miR-609 | 0.868644 | 3.74E-06 | 9.50E-05 | Up |
| hsa-miR-27a | 0.546001 | 5.68E-06 | 0.000136 | Up |
| hsa-miR-892b | 0.669767 | 1.38E-05 | 0.000269 | Up |
| hsa-miR-1283 | -1.16436 | 2.27E-14 | 1.96E-11 | Down |
| hsa-miR-31* | -0.87801 | 4.01E-09 | 8.64E-07 | Down |
| hsa-miR-518a-3p | -0.84251 | 5.45E-09 | 9.07E-07 | Down |
| hsa-miR-519e* | -0.87161 | 9.44E-09 | 1.16E-06 | Down |
| hsa-miR-488* | -0.80758 | 1.55E-08 | 1.65E-06 | Down |
| hsa-miR-566 | -0.83758 | 1.72E-08 | 1.65E-06 | Down |
| hsa-miR-1278 | -0.77114 | 2.70E-08 | 2.33E-06 | Down |
| hsa-miR-1291 | -0.7846 | 3.07E-08 | 2.41E-06 | Down |
| hsa-miR-515-5p | -0.80423 | 9.05E-08 | 6.51E-06 | Down |
| hsa-miR-591 | -0.7095 | 1.65E-07 | 1.09E-05 | Down |
