# Supplementary material for: Uncovering potential diagnostic biomarkers of acute myocardial infarction based on machine learning and analyzing its relationship with immune cells
Source: BMC Cardiovasc Disord. 2023 Jan 4;23:2. doi: 10.1186/s12872-022-02999-7 (PMC9814319; doi:10.1186/s12872-022-02999-7)
Supplement: Supplementary file 6 — Additional file 6: Table S3 hold-out validation results of each model. [file 12872_2022_2999_MOESM6_ESM.docx]

**Table S3 hold-out validation results of each model**

| **Classifier** | **Accuracy** | **Sensitivity** | **Specificity** | **AUC** |
| --- | --- | --- | --- | --- |
| Test1-Support vector machine (SVM) | 0.76 | 0.76 | 0.76 | 0.862 |
| Test1-Random forest (RF) | 0.88 | 0.88 | 0.88 | 0.934 |
| Test1-Decision tree (DT) | 0.80 | 0.80 | 0.80 | 0.863 |
| Test2-SVM | 0.878 | 0.875 | 0.880 | 0.930 |
| Test2-RF | 0.857 | 0.833 | 0.880 | 0.958 |
| Test2-DT | 0.714 | 0.708 | 0.720 | 0.763 |
